# Supplementary material for: Complementary Volume Electron Microscopy-based approaches reveal ultrastructural changes in germline intercellular bridges of D. melanogaster
Source: bioRxiv. 2025 Feb 23:2025.02.18.638836. Preprint. [Version 1] doi: 10.1101/2025.02.18.638836 (PMC11870571; doi:10.1101/2025.02.18.638836)
Supplement: 1 [file NIHPP2025.02.18.638836V1-supplement-1.pdf]

bioRxiv preprint doi: https://doi.org/10.1101/2025.02.18.638836; this version posted February 23, 2025. The copyright holder for this preprint (which was not certified by peer review) is the author/funder, who has granted bioRxiv a license to display the preprint in perpetuity. It is made available under aCC-BY 4.0 International license.

Figure S1. Sample preparation, acquisition, alignment, and analysis for different types of vEM. All described techniques are performed at room temperature with adaptations in preparation techniques based on the type of the sample. (A) For TEM, the samples are sectioned on an ultramicrotome with a diamond knife and transferred to support grids (round, 3mm diameter, thin metal foil). The maximum area of the sample is limited by these dimensions. The thickness of the sections varies between 50 and 300 nm and is defined by the penetration capacity of the electron beam. The resulting images provide a very high resolution (~ 0.2 nm), but higher magnification images result in a smaller field of view. (B) For FIB-SEM, minimal

bioRxiv preprint doi: https://doi.org/10.1101/2025.02.18.638836; this version posted February 23, 2025. The copyright holder for this preprint (which was not certified by peer review) is the author/funder, who has granted bioRxiv a license to display the preprint in perpetuity. It is made available under aCC-BY 4.0 International license.

manipulation is required before introducing the sample into the SEM sample chamber. The block surface is milled directly inside the SEM chamber and the images are collected from the newly exposed surface. The destructive nature of the acquisition prevents modification or reacquisition of the images once acquired. FIB removes 5-50 nm of the surface before capturing a SEM image, with the cycle continuing to create a nearly perfect 3D stack through a relatively small sample volume. The stack of images is easy to align, and isotropic resolution can be achieved. (C) For AT-SEM, blocks are sectioned using a diamond knife, creating a sequence of sections (arrays), which is transferred to a large support, such as a wafer or a coated glass coverslip. The physical sectioning by the diamond knife permits the processing of a relatively large area (> 1 mm^2) and a nearly unlimited z depth. The primary strength of the technique is the ability to use lateral screening of the arrays to efficiently locate the ROI, which can then be selectively imaged at higher resolution. With tiled acquisition there is no real upper limit to the area that can be acquired; further, sections are stable and can be repeatedly imaged at a range of resolutions. z resolution is limited by physical sectioning to ~50 nm. The alignment and segmentation steps of the workflow are frequently performed with the same program. The alignment can be performed automatically with the help of the program's algorithms or manually by adjusting the consecutive images individually. The segmentation depends on the scope of the desired model and the abundance and complexity of the structures; it can be done manually or with automation.

Supplemental Movie 1a. Movie of entire FIB-SEM image stack through the germline clusters within the germarium, which are rendered in Fig. 3.
https://doi.org/10.6084/m9.figshare.28430189

Supplemental Movie 1b. Movie of entire FIB-SEM image stack through part of the stage 4 egg chamber. https://doi.org/10.6084/m9.figshare.28430192

bioRxiv preprint doi: https://doi.org/10.1101/2025.02.18.638836; this version posted February 23, 2025. The copyright holder for this preprint (which was not certified by peer review) is the author/funder, who has granted bioRxiv a license to display the preprint in perpetuity. It is made available under aCC-BY 4.0 International license.

Supplemental Movie 2. Movie of a FIB-SEM serial section alignment through the ring canal presented in Fig. 2B, C. https://doi.org/10.6084/m9.figshare.28430066

bioRxiv preprint doi: https://doi.org/10.1101/2025.02.18.638836; this version posted February 23, 2025. The copyright holder for this preprint (which was not certified by peer review) is the author/funder, who has granted bioRxiv a license to display the preprint in perpetuity. It is made available under aCC-BY 4.0 International license.

Supplemental Movie 3a. A series of aligned sequential EM sections was obtained using FIB-SEM, focusing on the germline region. Different clusters were manually segmented with separate colors for each cluster. Ring canals for each cluster were generated using the isosurface feature of IMOD. https://doi.org/10.6084/m9.figshare.28430096

Supplemental Movie 3b. A series of sequential EM sections were obtained using FIB-SEM, focusing on the stage 2b purple cluster. The segmentation highlights the complexity of the fusome in the germline (white) and its interaction with the ring canals connecting different germline cells within the cluster. https://doi.org/10.6084/m9.figshare.28430117

Supplemental Movie 4. Aligned sequence of the RCs from the FIB-SEM dataset of the stage 4 EC shown in Fig. 4A. The inner rim of the ring canals is pseudocolored (green). https://doi.org/10.6084/m9.figshare.28430213

Fig. S4: Single-plane FIB-SEM images show the differences in ring canal size, thickness, and orientation within each cluster. Images were cropped to highlight the germline RCs within each cluster. (A) The two clusters from region 2b (purple and pink) were sufficiently covered to determine mitotic division of origin for all RCs. (B) For the stage 1 cluster (yellow), division of origin could be definitively assigned for about half of the RCs, and for the other half, origin could be narrowed down to one of two types. (C) The older, stage 4 EC (grey) was not

bioRxiv preprint doi: https://doi.org/10.1101/2025.02.18.638836; this version posted February 23, 2025. The copyright holder for this preprint (which was not certified by peer review) is the author/funder, who has granted bioRxiv a license to display the preprint in perpetuity. It is made available under aCC-BY 4.0 International license.

completely captured, so only the four posterior RCs directly connecting to the oocyte could be definitively characterized. Scale bars are 1 μm.

Supplemental Material 5. A composite of tiled images acquired with 5 nm resolution. Uncompressed image represents 1101x266 cm. https://doi.org/10.6084/m9.figshare.28430216

Supplemental Movie 5a. Aligned serial sequence of the OO-NC RC from the AT acquisition shown in Fig. 5G. https://doi.org/10.6084/m9.figshare.28430138

Supplemental Movie 5b. Aligned serial sequence of the NC-NC RC from the AT acquisition shown in Fig. 5H. https://doi.org/10.6084/m9.figshare.28430147

bioRxiv preprint doi: https://doi.org/10.1101/2025.02.18.638836; this version posted February 23, 2025. The copyright holder for this preprint (which was not certified by peer review) is the author/funder, who has granted bioRxiv a license to display the preprint in perpetuity. It is made available under aCC-BY 4.0 International license.

Supplemental Movie 5c. Aligned serial sequence of the OO-NC RC from the AT acquisition shown in Fig. 5G. The movie combines the EM sequence and the subsequent segmentation of the outline of the ring canal (green) and the portions of the interdigitations (light blue). https://doi.org/10.6084/m9.figshare.28430258

Supplemental Movie 6: Serial sections through the RC from the imperfectly prepared sample shown in Fig. 6. https://doi.org/10.6084/m9.figshare.28430162

bioRxiv preprint doi: https://doi.org/10.1101/2025.02.18.638836; this version posted February 23, 2025. The copyright holder for this preprint (which was not certified by peer review) is the author/funder, who has granted bioRxiv a license to display the preprint in perpetuity. It is made available under aCC-BY 4.0 International license.

Supplemental Movie 7a: Aligned serial sections of AT-SEM images of the anterior end of the germarium containing the germline stem cell niche. The movie combines the EM sequence and the subsequent segmentation. The image stack shows what appears to be a dividing germline stem cell; the two daughter cells are pseudo-colored.
https://doi.org/10.6084/m9.figshare.28430165

Supplemental Movie 7b: Aligned serial sections of AT-SEM images. The movie combines the AT-SEM sequence and the subsequent segmentation of the border cell cluster.
https://doi.org/10.6084/m9.figshare.28430177

Figure S1

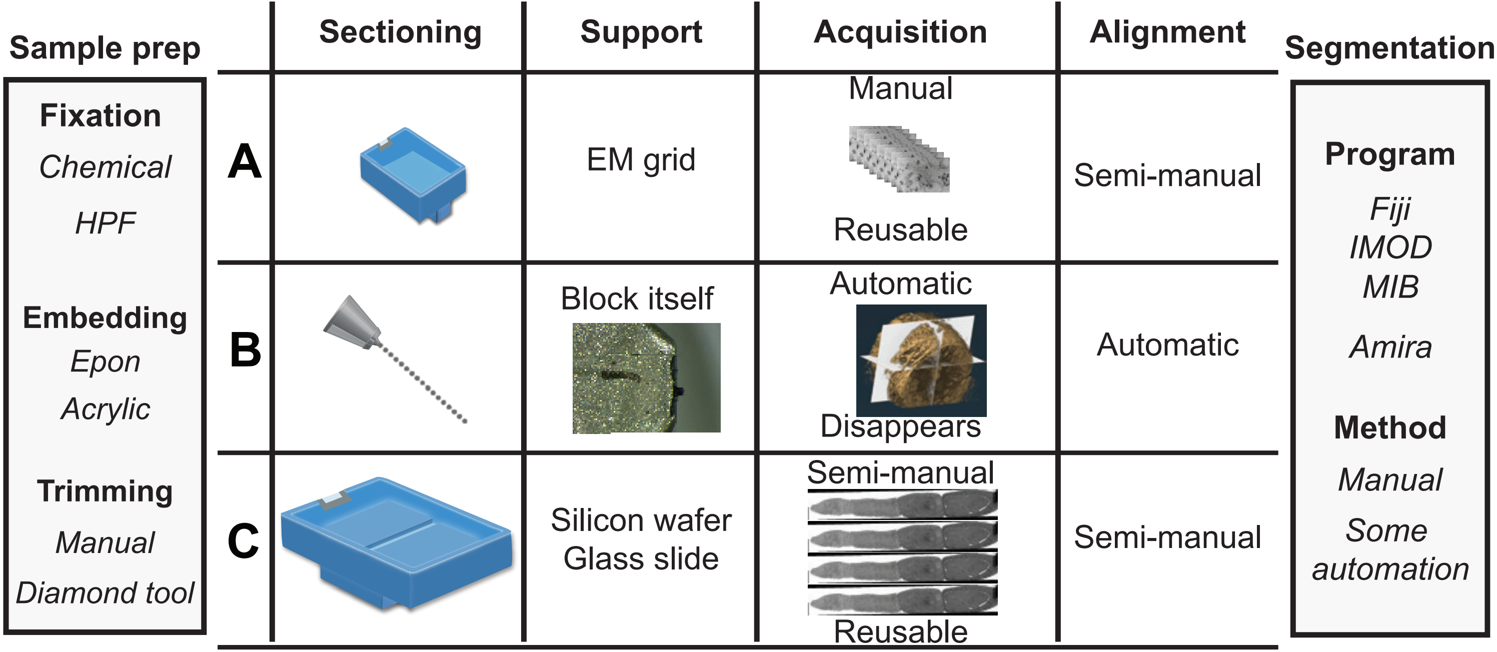

Sample prep
Fixation
Chemical
HPF
Embedding
Epon
Acrylic
Trimming
Manual
Diamond tool
Sectioning
Support
Acquisition
Alignment
A
EM grid
Manual
Reusable
Semi-manual
B
Block itself
Automatic
Disappears
Automatic
C
Silicon wafer
Glass slide
Semi-manual
Reusable
Semi-manual
Segmentation
Program
Fiji
IMOD
MIB
Amira
Method
Manual
Some automation

Figure S4

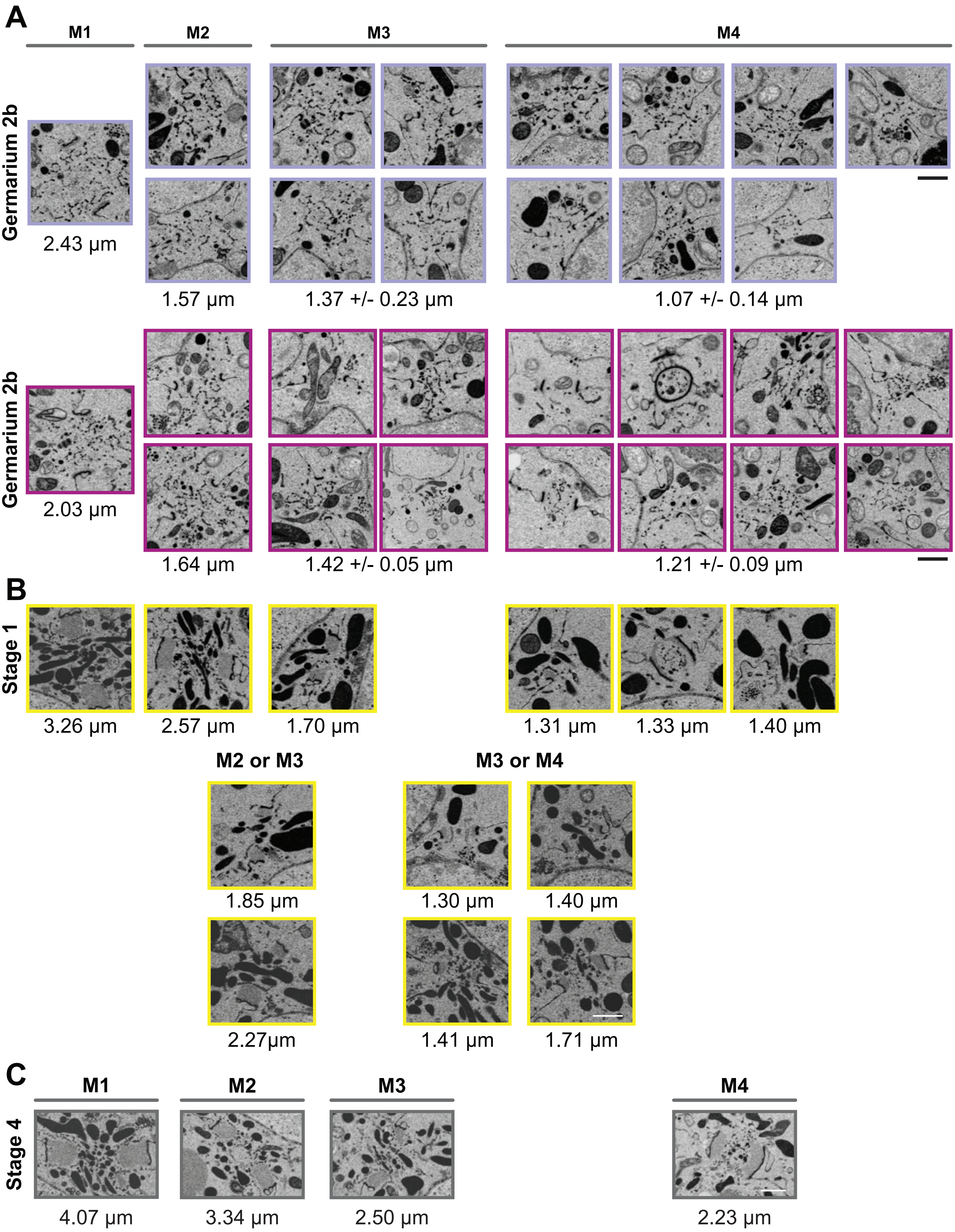
A
M1
M2
M3
M4
Germarium 2b
2.43 µm
1.57 µm
1.37 +/- 0.23 µm
1.07 +/- 0.14 µm
Germarium 2b
2.03 µm
1.64 µm
1.42 +/- 0.05 µm
1.21 +/- 0.09 µm
B
Stage 1
3.26 µm
2.57 µm
1.70 µm
1.31 µm
1.33 µm
1.40 µm
M2 or M3
M3 or M4
1.85 µm
1.30 µm
1.40 µm
2.27µm
1.41 µm
1.71 µm
C
M1
M2
M3
M4
Stage 4
4.07 µm
3.34 µm
2.50 µm
2.23 µm
